# Brain Functional Connectivity as a Mediator Between Hematological Metrics and Cognitive Decline in Children With Beta‐thalassemia Major

**DOI:** 10.1002/brb3.71363

**Published:** 2026-03-31

**Authors:** Shumin Xu, Yi Zheng, Yaowen Li, Xinyi Liu, Sixi Liu, Xiaodong Wang, Jiazhen Wu, Gui Huang, Mengting Liu, Hongwu Zeng

**Affiliations:** ^1^ Department of Radiology Shenzhen Children's Hospital Shenzhen China; ^2^ School of Biomedical Engineering Sun Yat‐sen University Shenzhen China; ^3^ Department of Radiology Jiangmen Central Hospital Jiangmen China; ^4^ Department of Electronic Engineering Shantou University Shantou Guangdong China

**Keywords:** beta‐thalassemia major, brain network, cognitive impairment, connectome‐based predictive modeling, fMRI, hematological abnormalities

## Abstract

**Purpose:**

This study aimed to identify functional brain connectivity patterns associated with cognitive performance in Beta‐thalassemia major (β‐TM) children and to determine whether hematological factors influence cognition indirectly through alterations in connectivity.

**Method:**

We recruited 25 children with β‐TM and 35 age‐matched healthy controls. Cognitive performance was assessed using the Wechsler Intelligence Scale (WIS). Resting‐state functional MRI data were processed to construct whole‐brain functional connectivity matrices. We applied network‐based statistics (NBS) to compare connectivity differences between groups and connectome‐based predictive modeling (CPM) with cross‐validation to predict cognitive scores. Mediation analyses were further conducted to test whether hematological metrics (hemoglobin level, red blood cell distribution width) impacted cognition through functional connectivity.

**Finding:**

Compared to controls, β‐TM children showed significantly reduced WIS scores and widespread disruptions in functional connectivity, particularly in cerebellar, motor, and temporal networks. The CPM approach identified a predictive network that largely overlapped with the NBS‐derived network and robustly predicted WIS scores. Mediation analysis revealed that hemoglobin and red blood cell distribution width influenced cognitive scores indirectly through altered connectivity, indicating a full mediation effect.

**Conclusion:**

This study provides evidence that hematological abnormalities in β‐TM children impair cognitive performance via their impact on functional brain networks. Functional connectivity signatures derived from CPM may serve as early neuromarkers of cognitive vulnerability and could inform future monitoring and intervention strategies in this population.

## Graphical abstract

Connectome predictive modeling (CPM) revealed reduced functional connectivity linked to cognitive underperformance in children with β‐thalassemia major (β‐TM), which appeared to be modulated by multiple hematological factors.

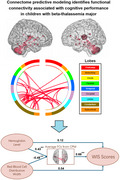

## 1 Introduction

Beta‐thalassemia major (β‐TM below), one of the most severe types of hereditary hemolytic anemia, has a global carrier rate of 1.5% (Galanello and Origa, 2010). The primary treatment for β‐TM requires frequent blood transfusions, which over time leads to iron accumulation in the blood, potentially damaging organs such as the heart, liver, and pancreas, and even causing brain damage (Berdoukas, Farmaki, Carson, Wood, and Coates, 2012; Manara et al., 2019). A landmark article published in 2019 recommended that all physicians consider cognitive impairment when treating β‐TM (Tartaglione et al., 2019). Other studies have also indicated that patients with β‐TM might experience brain iron overload due to long‐term transfusions, ineffective erythropoiesis, and increased intestinal iron absorption, leading to cognitive impairment (Monastero, Monastero, Ciaccio, Padovani, and Camarda, 2000; Nemtsas, Arnaoutoglou, Perifanis, Koutsouraki, and Orologas, 2015; Raafat et al., 2015). Most affected β‐TM patients experience cognitive function impairments, including difficulties in speech, executive function, working memory, and spatial perception (Pholngam et al., 2024; Tartaglione et al., 2019).

Studies using neuroimaging techniques have begun to uncover the neurological effects of β‐TM. These include silent ischemic lesions, white matter abnormalities, intracranial arterial stenosis, and irregularities in sensory evoked potentials (Karimi, Khanlari, and Rachmilewitz, 2008; Musallam et al., 2011; Nemtsas et al., 2015; Pazgal, Inbar, Cohen, Shpilberg, and Stark, 2016; Qiu et al., 2014; Raz, Koren, and Levin, 2019; Taher et al., 2006). In comparison to the structural abnormalities (Gosein et al., 2019; Gupta & Patel, 2008; Kim, Leung, Lerch, and Kassner, 2016; Kirk et al., 2009; Li et al., 2026), functional Magnetic Resonance Imaging (fMRI) offers more direct insights into brain function, shedding light on the neural mechanisms underlying cognitive impairments in β‐TM patients (Raz, Koren, Dan, and Levin, 2016). However, fMRI investigations of cognitive impairment in β‐TM remain limited.

It is noteworthy that the primary concern in patients with beta‐thalassemia who require medical attention are school‐aged children, where learning and memory impairments are significant issues in this population (Raafat et al., 2015). Indults, β‐TM–related injuries, are typically localized, with iron overload targeting deep subcortical structures like the basal ganglia, thalamus, and hippocampus (Akhlaghpoor et al., 2012; Manara et al., 2019). In contrast, the developing brain of children is more vulnerable. Chronic hypoxia in children with β‐TM leads to diffuse injury, affecting global regions. Although MRI studies in children with β‐TM are limited (Bu et al., 2023), research on other neurological disorders shows that children often experience more widespread neural damage compared to adults (Figaji, 2017; Parker, Donovan, Smith, and Noble‐Haeusslein, 2021), resulting in subtle and variable cognitive impairments that are difficult to detect with traditional, region‐specific methods. This increased vulnerability highlights the need for data‐driven approaches, such as whole‐brain connectomes, to detect subtle, widespread changes in brain function (Cheng et al., 2021; Rosenberg et al., 2016) in relation to cognitive task performance. These methods provide a more comprehensive understanding of how chronic hypoxia impacts the developing brain in children with β‐TM.

Connectome predictive modeling (CPM) methods, which utilize machine learning algorithms and include a cross‐validation step (Scheinost et al., 2019; Yip, Kiluk, and Scheinost, 2020), have increasingly been used to predict behavioral phenotypes from large‐scale functional networks (Finn et al., 2015; Ibrahim et al., 2022; Yip, Scheinost, Potenza, and Carroll, 2019). This data‐driven method shows particular promise for moving beyond group comparisons to identify disruptions in functional interactions associated with cognitive task performance, potentially aiding in the discovery of brain‐behavior associations and individual‐level biomarkers (Cheng et al., 2021; Liu, Backer, Amey, Splan, et al., 2021). So far, no study has yet examined whether the functional organization of the brain can predict cognitive deficit in children with β‐TM. Our study is the first to employ a CPM approach to identify neural markers of childhood β‐TM.

As one of the most oxygen‐demanding organs in the body, the brain is highly sensitive to disruptions in its oxygen supply. In β‐TM patients, the iron overload caused by repeated blood transfusions might impair oxygen delivery and metabolism in the brain, thereby contributing to cognitive dysfunction. Although direct investigations in β‐TM are lacking, research on conditions such as hypoxic‐ischemic encephalopathy (HIE) has demonstrated that oxygen deprivation disrupts metabolism in the fronto‐parietal cortex, temporal cortex, and cerebellum—key regions supporting cognitive function. PET imaging studies have revealed that these regions are particularly vulnerable to hypoxia‐induced damage. For example, research by He et al. (2022) demonstrated reduced metabolic connectivity in the fronto‐parietal cortex in patients with HIE, while Reed et al. (1999) and Di Paola et al. (2008) found significant hypometabolism in the temporal cortex, especially the hippocampus, in hypoxic patients with amnesia. These findings suggest that β‐TM patients may experience similar metabolic disruptions, resulting in cognitive impairments due to chronic oxygen deprivation.

In this study, we hypothesize that data‐driven approaches such as Connectome‐based Predictive Modeling (CPM) can help identify reliable patterns of functional brain connectivity that contribute to cognitive decline in children with β‐TM. Given the chronic oxygen and metabolic disruptions associated with the disease, regions such as the fronto‐parietal cortex, temporal lobe, and cerebellum are likely to be critically involved. We further propose that abnormal functional connectivity may be driven, at least in part, by hematological metrics. To test these hypotheses, we designed a coherent analytical framework. First, we applied CPM to whole‐brain functional connectivity data to identify networks predictive of cognitive performance. Next, we used mediation analysis to examine whether the impact of hematological factors on cognition is mediated through alterations in brain network connectivity. We hypothesize that the mechanisms underlying cognitive deficits in β‐TM differ from those in healthy controls, with hematological pathologies modulating network connectivity and, consequently, cognitive function. A central objective is to determine whether these neural markers can account for the variability in clinical outcomes observed among patients. By integrating CPM with mediation analysis, this study establishes a novel data‐driven framework for elucidating the neural mechanisms of cognitive impairment in β‐TM and the complex interplay between brain connectivity and hematological factors.

## 2 Materials and Methods

### 2.1 Ethics Approval Disclosure Statement

This study was approved by the Ethics Committee of Shenzhen Children's Hospital (Approval No. 202004002). All procedures involving human participants were conducted in accordance with the ethical standards of the institutional and national research committee and with the 1964 Helsinki Declaration and its later amendments. Written informed consent was obtained from all participants’ legal guardians, and assent was also obtained from all child participants, prior to exerting influence to participate in the study. All data were fully de‐identified to protect participant privacy and were handled in accordance with the protocols of our institution.

### 2.2 Subjects

From May 2020 to October 2023, we recruited a total of 27 patients with beta‐thalassemia and 38 healthy controls from our institutional referral centers for beta‐thalassemia. Two β‐TM patients and three healthy subjects were excluded due to image artifacts (*n* = 3) and significant registration errors (n = 2). The final analysis included 25 β‐TM patients (mean hemoglobin level: 104.28 ± 13.31 g/L; red blood cell distribution width: 42.06 ± 9.21 g/L), all recruited from the Pediatric Hematology Unit at Shenzhen Children's Hospital. A priori power analysis was not conducted due to the challenges inherent in recruiting pediatric patients with beta‐thalassemia major.

Exclusion criteria for participants were (1) presence of other mental disorders, personality disorders, or psychotropic drug dependence; (2) inability to cooperate during MRI examination, resulting in poor image quality; (3) history of other organic or metabolic brain diseases; and (4) other MRI contraindications.

Healthy control participants were primarily recruited from the relatives or acquaintances of the patients. None of the control subjects were recruited from a hospital setting; all were enrolled from individuals attending routine health examinations. Eligibility required no prior history of hematological or chronic diseases, no clinical signs or symptoms suggestive of anemia, and confirmation of good overall health status by physicians at the time of enrollment. Complete blood count (CBC) data were not specifically obtained to prevent unnecessary invasive procedures in healthy volunteers, as such testing was not clinically indicated in the absence of pathological signs. These strict inclusion criteria ensured that subjects with anemia or other relevant conditions were effectively excluded. Furthermore, to minimize environmental or dietary confounding, healthy controls were primarily recruited from the same local communities and households as patients, ensuring comparable socio‐demographic exposure. Although individual diet was not directly assessed, this recruitment strategy reduced variability attributable to lifestyle factors.

### 2.3 Clinical Cognitive Function Assessment

We conducted behavioral tests on both healthy children and those with illnesses, using the latest version (Edition IV) of the Wechsler Intelligence Scale (WIS). This test covers four areas: verbal comprehension, perceptual reasoning, working memory, and processing speed. For our analysis, the WIS scores are calculated as the sum of the scores from these four areas.

### 2.4 Image Acquisition

Structural brain imaging data were obtained on a 3‐Tesla MRI unit (Magnetom Skyra, Siemens Medical Solutions, Erlangen, Germany) with a 20‐channel head coil array, located at Shenzhen Children's Hospital. A T1‐weighted three‐dimensional magnetization prepared‐rapid gradient echo (3D‐MPRAGE) sequence was acquired with the following parameters: 176 contiguous sagittal slices, 1 mm × 1 mm × 1 mm raw voxel size, repetition time (TR) = 2300 ms, echo time (TE) = 2.26 ms, field of view (FOV) = 256 mm, number of averages = 1, and slice oversampling = 18.2%. Besides, a T2‐weighted sequence with TR = 2300 ms and TE = 10.6 ms was performed before T1‐weighted scanning to exclude organic cerebral lesions.

fMRI data was obtained by using an echo‐planar imaging (EPI) sequence with the following parameters: repetition time = 2000 ms, echo time = 30 ms, flip angle = 90 °, slice thickness/gap = 4.0/0.0 mm, matrix = 64 × 64, field of view = 24 × 24 cm^2^, 32 axial slices. 130 volumes.

All participants were asked to lie still and stay awake with their eyes closed during scanning. A surveillance camera was set to monitor the subjects for fear of accidents. Foam pads were used to restrain head movements, and earplugs were added to drown the scanner noise. Once motion artifacts were presented, the subject was requested to receive MRI rescanning immediately to ensure better image quality.

### 2.5 Imaging Preprocessing

Functional imaging data were processed using SPM12 software (https://www.fil.ion.ucl.ac.uk/spm/). For each participant, the initial 10 volumes were discarded to achieve dynamic equilibrium and adaptation to the scanning conditions. The remaining functional images were then adjusted for slice timing to correct time delays between slices. Head motion was corrected using a six‐parameter rigid body transformation during the realignment analysis. Participants with head motion exceeding 3 mm in translation or 1.5° in rotation were excluded from further analysis; however, no participants met these exclusion criteria in this study. Each participant's structural image was coregistered to the mean functional image and segmented into gray matter, white matter (WM), and cerebrospinal fluid (CSF) for normalization. All functional images were spatially normalized to the standard Montreal Neurological Institute (MNI) space with an isotropic voxel size of 3 mm. To enhance the signal‐to‐noise ratio, the normalized functional images were smoothed using a 4 mm full‐width at half‐maximum Gaussian filter. A linear detrending and temporal bandpass filtering (0.01 Hz to 0.08 Hz) procedure was applied to mitigate the effects of low‐frequency drift and high‐frequency physiological noise. Additionally, several sources of spurious variance, along with their temporal derivatives, were addressed using linear regression to reduce physiological noise and remove artifacts. This included averaged signals from WM, CSF, and six head motion parameters.

### 2.6 Functional connectivity construction

To construct the whole‐brain functional connectivity (FC) matrix, the Anatomical Automatic Labeling (AAL) template was employed to segment the brain into 116 regions of interest (ROIs), comprising 78 cortical, 12 subcortical, and 26 cerebellar regions (Tzourio‐Mazoyer et al., 2002). For each ROI, a representative time series was obtained by averaging the time series of all voxels within the region. Pearson's correlation analysis was then conducted between each pair of ROIs to determine the correlation coefficients, which were subsequently normalized using a Fisher z‐score transformation. This process resulted in a symmetric functional connectivity matrix (116 × 116) for each participant. The upper triangular portion of the adjacency matrix was extracted and converted into a vectorized feature space consisting of 6670 dimensions.

### 2.7 Network Based Statistics (NBS)

To identify specific pairs of brain regions with altered functional connectivity in cirrhotic patients, we used the network‐based statistic (NBS) approach (Zalesky, Fornito, and Bullmore, 2010). First, we conducted two‐sample one‐tailed *t*‐tests on each connection that was significantly nonzero (p < 0.05, Bonferroni corrected) in at least one participant. A primary threshold (p < 1e‐4 in this study) (Liu, Backer, Amey, and Forbes, 2021; Wang et al., 2013) was then applied to define a set of suprathreshold links, within which connected components and their sizes (number of links in these components) were determined.

To estimate the significance of each component, a null distribution of connected component size was empirically derived using a nonparametric permutation approach with 10,000 permutations. For each permutation, subjects were randomly reassigned into two groups, and two‐sample one‐tailed *t*‐tests were performed on the same set of connections. The same primary threshold (p < 1e‐4) was used to generate suprathreshold links, and the size of the maximal connected component was recorded. The corrected p‐value for a connected component of size M (number of edges) found in the right grouping of control subjects and patients was determined by calculating the proportion of the 10,000 permutations where the maximal connected component size was larger than M.

NBS was applied to compare functional connectivity (FC) differences between control subjects and β‐TM patients, revealing the disrupted connectivity induced by β‐TM.

### 2.8 Connectome‐based Predictive Modeling (CPM)

CPM is a powerful technique in neuroscience that seeks to predict individual differences in cognitive, behavioral, or clinical outcomes based on patterns of brain network connectivity (Rosenberg et al., 2016; Shen et al., 2017). The first step in the CPM process involves identifying the relevant networks for prediction. To do this, we calculated Pearson's correlation between each edge in the connectivity profile and the WIS score for every participant. The resulting correlation values were thresholded at *p* < 0.005 (Wu et al., 2023). This allowed us to distinguish a positive network (edges with positive correlations to WIS scores) and a negative network (edges with negative correlations).

For each participant, we calculated the “network strength” by summing the edges in the positive and negative networks separately, resulting in a weighted degree measure for both networks. To generate predicted compulsive behavior scores, we used leave‐one‐out cross‐validation (LOOCV). In each iteration, the data from N‐1 participants were used to train the model, where N represents the total number of participants. The positive and negative networks were derived from the training set, and network strengths were computed for each participant. Two separate predictive models were created to predict WIS scores based on the positive and negative networks. A simple linear model was fitted to relate network strengths (independent variable) to WIS scores (dependent variable). Additionally, a combined model was constructed, which incorporated both the positive and negative networks. This model used a general linear model (GLM) to simultaneously consider the strengths of both networks. The three models were then applied to novel participants’ network strengths to generate predicted scores. We ran the models independently for all participants (including both β‐TM and healthy controls), resulting in individualized predicted scores. It is important to note that the networks and predictive models varied slightly across iterations, as the training set for feature selection differed in each N‐1 participant group. Thus, we identified the intersection of the networks across all iterations to define the final positive and negative networks.

### 2.9 Localization of Predictive Networks

Predictive networks were summarized at various levels of data reduction, including edge, node, and network levels (Yip et al., 2020). The overlap of nodes with macroscale brain regions (such as the motor cortex and cerebellum) was determined based on anatomical labels from Finn et al. (2015). The overlap of nodes with canonical functional network localizations (like frontoparietal and sensorimotor networks) was based on the functional networks from Nobel et al. (2017). Additionally, for each node, the network theory measure “degree” was calculated as the sum of the number of edges for each node within the predictive networks.

Visualizations of predictive edges were created using BioImage Suite Web (https://bioimagesuiteweb.github.io/alphaapp/index.h%B2‐TMl) (Papademetris et al., 2006). High‐degree nodes were defined as the top nodes with the most edges or connections across all iterations of the predictive model.

### 2.10 Significance of CPM Performance

For connectome analyses, the correspondence between predicted and actual values, or model performance, was assessed using Pearson's correlation. Negative correlations were set to zero. During cross‐validation, analyses in the left‐out folds are not independent, leading to an overestimation of degrees of freedom for parametric *p*‐values. Therefore, permutation testing was performed. To generate null distributions for significance testing, we randomly shuffled the correspondence between behavioral variables and connectivity matrices by permuting subject assignments for behavioral variables 1000 times and reran the CPM analysis with the shuffled data. Based on these null distributions, the *p*‐values for predictions were calculated (Lake et al., 2019). Given our hypothesis of a positive association between predicted and actual values, one‐tailed *p*‐values are reported.

### 2.11 Follow‐up Analyses

To determine the construct specificity of high‐degree nodes in predicting WIS scores, follow‐up tests were carried out by retaining the high‐degree nodes and all edges connected to them (i.e., removing all other edges). This approach aimed to assess the robustness of these networks in predicting WIS scores.

### 2.12 Mediation Analysis

The cognitive deficits observed in β‐thalassemia major (β‐TM) are commonly attributed to brain iron overload and associated hematological abnormalities. However, the specific role of brain function in this process remains poorly understood. We hypothesize that brain function may serve as a mediator between hematological abnormalities and cognitive impairments. To test this hypothesis, we conducted a causal mediation analysis (Tingley, Yamamoto, Hirose, Keele, and Imai, 2014) to investigate whether functional connectivity, as identified through the CPM method, mediates the relationship between hypothesized hematological risk factors (i.e., hemoglobin level and red blood cell distribution width) and cognitive performance. Age, gender, and brain volume were included as covariates. Using Hayes' PROCESS macro for SPSS (Model 4), we estimated both direct and indirect effects while adjusting for the aforementioned covariates across all pathways. The significance of the indirect effect was tested using bootstrapping with 1000 resamples, and 95% confidence intervals (CIs) were generated for the mediation effect. Continuous variables were mean‐centered to mitigate multicollinearity, and the p‐values from multiple comparisons were corrected using the false discovery rate (FDR) method. The significance threshold for all analyses was set at 0.05.

## 3 Results

### 3.1 Demographic information and clinical variables

In total, 25 individuals with β‐TM were included in the study, consisting of 15 males with an average age of 9.83 years (standard deviation ± 2.09 years). For the normal control group, 35 individuals were included in the study, consisting of 19 males with an average age of 9.72 years (standard deviation ± 1.81 years). Comprehensive demographic and clinical details of these groups are provided in Table 1.

**TABLE 1:** Demographic and clinical characteristics for β‐TM and NC subjects

Characteristic	β‐TM	NC
Demographic		
Subjects (*n*)	25	35
Sex: male/female (*n*)	13/12	19/16
Age (years)	9.83 ± 2.09 (6.33–15.08)	9.72 ± 1.81(6.42–13.08)
Clinical measurements		
Wechsler Intelligence Scale	71.57 ± 16.25 (35–106)	107.07 ± 11.23(88–127)
Hemoglobin (g/L)	104.28 ± 13.31 (82–126)	N/A
RDW (fL)	42.06 ± 9.21(13.70–63.10)	N/A

*Note*: Data are presented as mean ± standard deviation (range) unless otherwise specified.

Abbreviations: β‐TM, beta‐thalassemia major; NC, normal control; RDW, red cell distribution width.

### 3.2 Between Group Differences in Clinical Measurements

Overall, individuals with β‐TM exhibited significantly lower WIS scores (p < 0.001) compared to normal controls, suggesting impaired cognitive function in β‐TM patients. Detailed information on the WIS scores for these groups is presented in Table 1.

Hematological metrics such as hemoglobin level and red blood cell distribution width (RDW) were exclusively measured in β‐TM patients in this study. In β‐TM patients, WIS scores didn't show negative correlations with hematological metrics (p's > 0.36).

### 3.3 Disrupted Functional Connectivity in β‐TM

**FIGURE 1:**
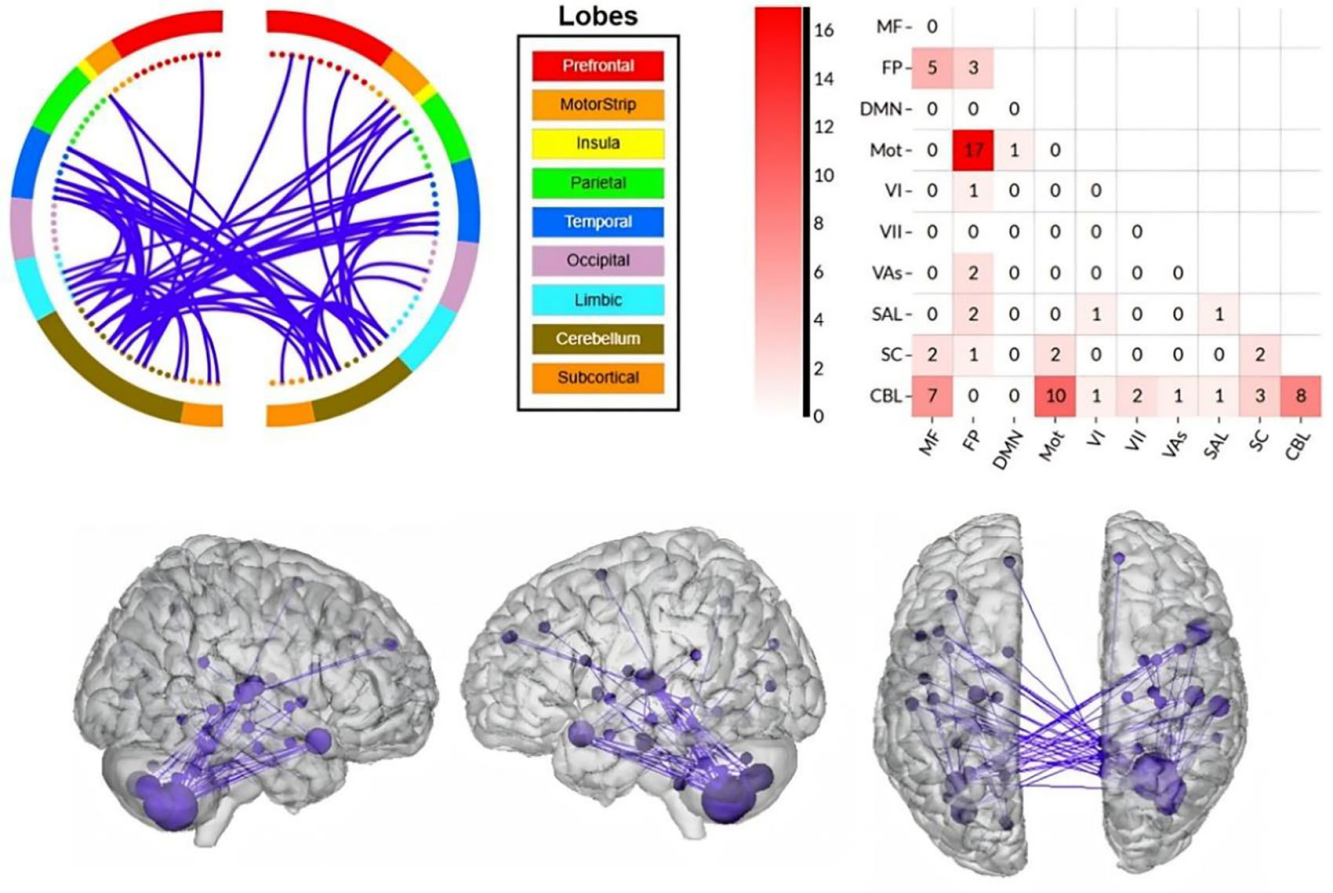
Brain‐wide functional connectivity impairment in β‐TM. β‐TM patients exhibit a large number of decreased/impaired functional connectivity across a wide range of the brain regions (blue edges). No increased functional connectivity was found in β‐TM patients. Larger spheres indicate nodes with more edges, and smaller spheres indicate nodes with fewer edges. Within‐ and between‐network connectivity for the impaired functional networks are summarized using canonical networks. Cells represent the total number of edges connecting nodes within and between each network, with darker colors indicating a greater number of edges.

NBS was initially applied to compare functional connectivity (FC) differences between control subjects and β‐TM patients. Using a cluster‐defining threshold of p < 1e‐4 (as explained in Materials and Methods), a single network consisting of 72 connections among 41 brain regions was identified, showing decreased functional connectivity in the β‐TM group (p = 0.012). This decreased connectivity was primarily found among the cerebellum's posterior lobules, including Crus II–VIIb, cerebellum X, cortical regions in motor areas such as Heschl's gyrus (HG) and the superior temporal gyrus (STG) (Figure 1). The top five regions found with the highest degrees are the right cerebellum XIIb, the right cerebellum XIII, the left and the right cerebellum Curs II, and right superior temporal cortex.

### 3.4 Prediction of WIS Score Using CPM

**FIGURE 2:**
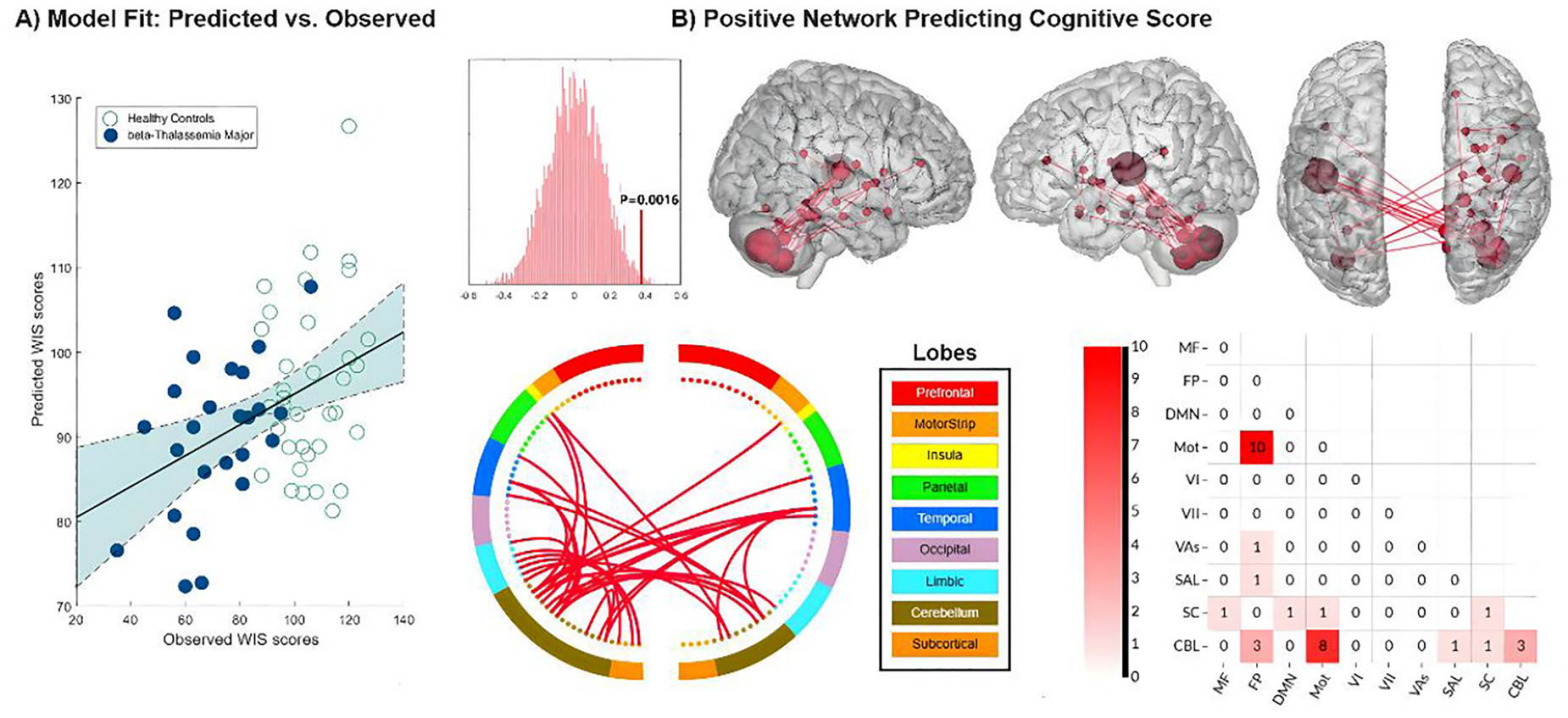
Brain‐wide functional connectivity predicts cognitive task performance. **(A)** The scatter plot shows a significant positive correlation between observed WIS scores (x‐axis) and scores predicted from whole‐brain functional connectivity (y‐axis), demonstrating the Connectome‐based Predictive Model's (CPM) predictive accuracy (r = 0.30, p = 0.016, permutation testing). Each dot represents an individual participant, with β‐TM patients shown in blue and healthy controls in white. The inset histogram displays the null distribution of correlations from 1000 permutations, with the red line indicating the observed correlation. **(B)** Brain regions and connections composing the positive network are visualized. In this network, stronger functional connectivity (represented by red lines) is associated with higher WIS scores. None of the connectivity in the negative network of the CPM model was significant in this study. Spheres are color‐coded by brain lobe, and the size of each node is proportional to its number of connections within the predictive network.

The comprehensive CPM model demonstrated that brain‐wide connectivity patterns could predict cognitive task performance, with only positive networks consisting of 93 connections significantly identified (r = 0.30, p = 0.016 via permutation testing) (Figure 2A). To ensure the robustness of our findings, we performed follow‐up regression analyses to assess the impact of potential covariates. The CPM model remained a significant predictor of WIS scores even after separately controlling for key demographic variables in the regression models, including age (r = 0.27, p = 0.021), sex (r = 0.28, p = 0.019), and group status (β‐TM vs. healthy control) (r = 0.27, p = 0.022). Further analyses were conducted using ten‐fold cross‐validation, yielding similar results; however, as anticipated, the correlation coefficient was slightly lower with ten‐fold cross‐validation compared to leave‐one‐out cross‐validation (r = 0.25, p = 0.031).

### 3.5 Network Anatomy and Localization of Circuits

Figure 2B provides a detailed overview of the cognitive networks, where only positive networks were detected, with no connectivity observed in negative networks. The highest‐degree nodes (those with the most connections) in the positive network were predominantly located in the posterior lobules of the cerebellum, including Crus II–VIIb and cerebellum VIII, as well as cortical regions in motor areas such as Heschl's gyrus (HG) and the superior temporal gyrus (STG), among others. These findings are notably similar to those shown by Network‐Based Statistic (NBS) analysis. The top four regions with the highest degree of connectivity were the left Heschl cortices, left and right cerebellum XIIb, and right cerebellum XIII. On a network level, the overall between‐network connectivity was primarily characterized by interactions between the frontoparietal and motor networks, as well as connections among the cerebellum and motor networks.

### 3.6 Follow‐up Analyses

**FIGURE 3:**
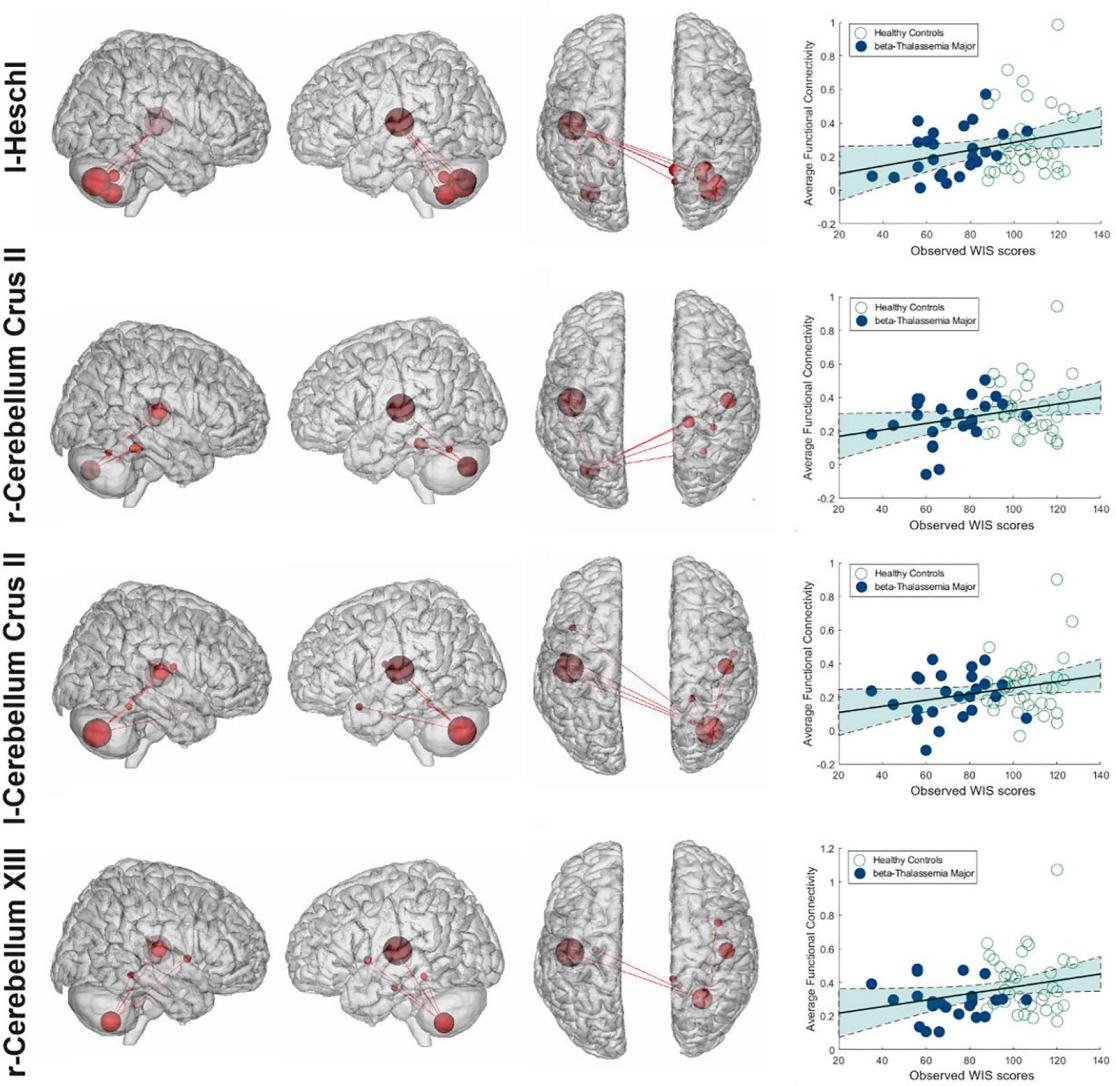
Sensitivity Analysis of High‐Degree Nodes in Predicting Cognitive Scores. This figure illustrates a follow‐up analysis to confirm the importance of the four highest‐degree nodes from the predictive model, which include the l‐Heschl, r‐Cerebellum Crus II, l‐Cerebellum Crus II, and r‐Cerebellum XIII. For each of these four nodes, we isolated all of its connections and removed the rest of the network. The scatter plots on the right show that the average functional connectivity of each of these individual sub‐networks still significantly correlates with the observed WIS scores. This demonstrates that these specific brain regions are robust drivers of the overall model's predictive power.

Follow‐up analyses examined the sensitivity of high‐degree nodes (i.e., most predictive features) in forecasting WIS scores (Figure 3). We preserved the high‐degree nodes from CPM and all their connected edges while removing all other edges. After FDR correction and adjusting for age, sex, brain volume, and group status, the top four high‐degree nodes from the CPM network still show significant predictive power for the WIS score (r = 0.24, *p*‐FDR = 0.039 for left Heschl cortices; r = 0.24, *p*‐FDR = 0.041 for right cerebellum XIIb; r = 0.23, *p*‐FDR = 0.046 for right cerebellum XIIb; and r = 0.22, *p*‐FDR = 0.048 for right cerebellum XIII.)

### 3.7 Predictive Power Between β‐TM and Controls

**FIGURE 4:**
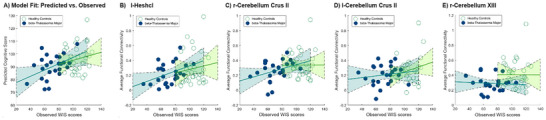
Separate regression analyses were performed for the β‐TM and control groups independently. **(A)** The predictive strength of the full CPM network was significantly correlated with WIS scores only in the β‐TM group (solid blue line), not in controls (dashed green line). **(B–E)** This specificity was also observed for the sub‐networks derived from the high‐degree nodes. Significant relationships with cognitive task performance were identified exclusively in β‐TM patients, regardless of the networks identified in CPM or the sensitivity of high‐degree nodes.

To determine whether the identified network serves as a generalized predictor of cognitive task performance for all participants or specifically indicates cognitive deficits in β‐TM patients, we conducted separate regression analyses using the network strength with WIS scores for both β‐TM and control groups. The results indicated that the network was predictive only for β‐TM children (r = 0.30, p = 0.031; Figure 4A), and not for the control group. Further analyses revealed that the node degree for the left Heschl's gyrus (r = 0.29, p = 0.035) and the right Cerebellum Crus II (r = 0.27, p = 0.042) were significant predictors of WIS scores in β‐TM children. However, none of the node degrees in these regions predicted WIS scores in healthy controls.

### 3.8 Association of Task Performance, Functional Network Strength and Hematological Metrics

**FIGURE 5:**
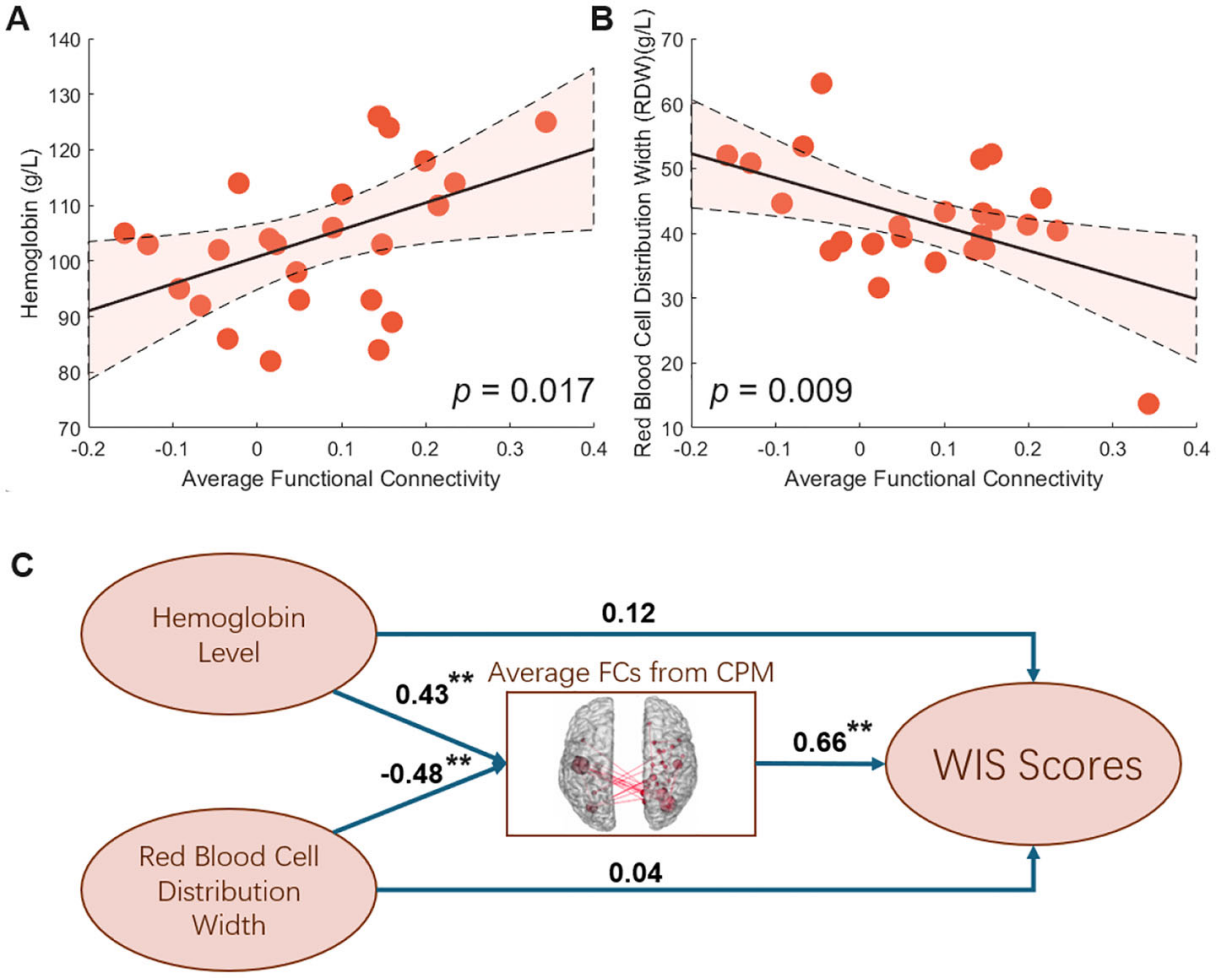
Univariate analyses revealed that higher **(A)** hemoglobin levels and **(B)** lower red blood cell distribution width were linked to increased functional connectivity, as identified by CPM. **(C)** SEM suggests that the functional connectivity observed in CPM mediates the impact of hematological abnormalities on WIS scores.

After FDR correction and adjusting for age, sex, and brain volume, univariate analyses showed that higher hemoglobin levels (r = 0.32; *p*‐FDR = 0.017, Figure 5A) and lower red blood cell distribution width (r = 0.35; *p*‐FDR = 0.009, Figure 5B) were associated with increased functional connectivity as identified by CPM.

The mediation analysis revealed that hemoglobin level had a significant positive effect on BAI (a = 0.43, SE = 0.12, p = 0.0088). The results also showed that red blood cell distribution width had a significant negative effect on BAI (a = −0.48, SE = 0.07, p = 0.0031), indicating that higher red blood cell distribution widths were associated with lower (more negative) functional connectivity within the CPM network. In turn, average functional connectivity within the CPM network significantly predicted the WIS scores (b = 0.66, SE = 0.15, p = 0.001). The direct effect of hemoglobin level (c′ = 0.12, SE = 0.08, p = 0.37) and red blood cell distribution width (c′ = 0.04, SE = 0.09, p = 0.80) on WIS scores was not significant, suggesting that the direct relationship between hemoglobin level, red blood cell distribution width, and WIS scores is weak. Critically, the indirect effect of hemoglobin level (a × b = 0.39, 95% CI [0.29, 0.55]) and red blood cell distribution width (a × b = −0.44, 95% CI [−0.57, −0.27]) on WIS scores through average functional connectivity within the CPM network was significant, indicating full mediation. This finding suggests that the effect of hemoglobin level and red blood cell distribution width on WIS scores operates primarily through its influence on average functional connectivity within the CPM network.

## 4 Discussion

In this study, we investigated the impairment of functional connectivity in β‐TM children using comprehensive whole‐brain functional connectivity comparisons and connectome‐behavior predictive mapping approaches. Our findings revealed that the impaired functional connectivity in β‐TM may influence cognitive performance.

### 4.1 Novel Insights From fMRI in Children With β‐TM

The use of fMRI‐derived connectivity markers in β‐TM offers important clinical advantages over structural imaging and neuropsychological testing alone. Structural MRI primarily reveals overt lesions or iron deposition, which often appear only after substantial damage has occurred. Likewise, cognitive scales capture the functional outcome but are insensitive to subtle or early neural disruptions and may be influenced by educational or cultural factors. By contrast, functional MRI provides a direct window into the brain's dynamic organization, allowing detection of diffuse connectivity alterations that precede gross structural change and are difficult to quantify through behavioral testing.

Our findings demonstrate that functional connectivity patterns not only differentiate β‐TM children from controls but also mediate the relationship between hematological abnormalities and cognitive performance. This mediation highlights a mechanistic pathway through which systemic disease burden translates into neural dysfunction, providing insight unattainable from blood tests or neurocognitive scales alone. In practice, fMRI‐based connectivity markers could serve as early biomarkers to identify children at greatest risk for cognitive decline, enabling timely educational support, targeted therapies, or adjustments in transfusion and chelation regimens before irreversible impairment develops.

Furthermore, these neuromarkers offer an objective tool for monitoring treatment efficacy. For example, functional connectivity strength could be tracked longitudinally to evaluate whether interventions—ranging from optimized hematological management to novel neuroprotective or cognitive rehabilitation strategies—restore or stabilize brain network integrity. Because the predictive network we identified was specific to β‐TM patients rather than controls, it holds promise as a disease‐relevant biomarker that reflects the unique neurocognitive vulnerability of this population.

### 4.2 Cognitive and Functional Impairments in β‐TM

#### 4.2.1 Cognitive Impairment in β‐TM

We observed a notable reduction in cognitive abilities in β‐TM children, as measured by the Wechsler Intelligence Scales. Children with β‐TM are susceptible to excessive iron accumulation in the brain, resulting from chronic blood transfusions, ineffective erythropoiesis, and increased intestinal iron absorption. This accumulation may contribute to cognitive deficits (Manara et al., 2019).

#### 4.2.2 Disrupted Functional Connectivity in β‐TM

In the imaging studies, notable findings emerged from the NBS‐based comparison and post‐hoc analysis among groups. β‐TM patients displayed widespread disruptions in functional connectivity compared to control subjects, indicating more severe brain impairment due to β‐TM. Notably, no significant increases in functional connectivity were observed in β‐TM children. The disrupted functional connectivity primarily occurred between the motor network and fronto‐parietal networks, as well as between the cerebellum and fronto‐parietal networks, the cerebellum and motor networks, and within the cerebellum network itself.

#### 4.2.3 Fronto‐Parietal Network Contributions

It's worth noting that the term frontoparietal network here refers not only to the cortical regions of the frontal and parietal lobes, but also to cerebellar regions associated with this network, particularly the posterior cerebellar lobeles, including Crus II–VIIb (Li et al., 2013; Noble et al., 2017). It is involved in a number of non‐motor, cognitive, and executive functions. These functions include working memory, planning, organizing, and strategy formation, which are important for creative divergent thinking (Prati, Pontes‐Silva, and Gianlorenço, 2024; Liu, Amey, and Forbes, 2017; Forbes and Liu, 2026).

#### 4.2.4 Temporal Cortex Contributions

When referring to the motor cortex, we primarily mean the Heschl cortex and the superior temporal cortices. Both Heschl's gyrus (HG) and the superior temporal gyrus (STG) are crucial for auditory processing and language (Fernández, Velásquez, Porrero, de Lucas, and Martino, 2020). Heschl's gyrus, also known as the transverse temporal gyrus, is a significant component of the posterior portion of the STG and serves as the primary auditory area. It is involved in various functions, including speech production, phonologic retrieval, semantic processing, and language comprehension (Mustroph, Zekelman, and Golby, 2020). Superior Temporal Gyrus (STG) is divided into anterior, middle, and posterior parts, encompassing HG and Wernicke's area. Bilateral activation of the STG enables the perception of speech sounds of varying lengths, such as syllables, words, and pseudowords (Friederici, Rüschemeyer, Hahne, and Fiebach, 2003). Damage to the STG on both sides can result in pure word deafness, highlighting its role in mapping acoustic signals to abstract speech. Additionally, the STG is essential for short‐term auditory sensory memory. Functional connectivity between these regions facilitates the integration of perceptual information with cognitive functions. Disruptions in this connectivity can result in difficulties processing and understanding perceptual information, thereby impairing overall cognitive performance.

#### 4.2.5 Cerebellar Cortex Contributions

Recent anatomical and functional studies have highlighted that various regions of the cerebellum are involved in a broad spectrum of cognitive functions beyond its traditional role in sensorimotor control (King, Hernandez‐Castillo, Poldrack, Ivry, and Diedrichsen, 2019; Strick, Dum, and Fiez, 2009). The cerebellum has been implicated in executive function (Koziol, Budding, & Chidekel, 2012), attention (Osaka et al., 2004), and emotional processing (Guell, Gabrieli, and Schmahmann, 2018). Specifically, cerebellum lobe X, a crucial hub in the PMN, has been identified as a non‐motor area in recent research (Guell and Schmahmann, 2020) and is thought to be associated with visual working memory and visual recognition (King et al., 2019). The exact role of the cerebellum in cognitive performance for β‐TM patients warrants further investigation in future studies.

### 4.3 Functional Decoding of WIS Scores in β‐TM

The Wechsler Intelligence Scale includes four aspects: Verbal Comprehension Index, Perceptual Reasoning Index, Working Memory Index, and Processing Speed Index, which is a comprehensive evaluation of the cognitive functions of children with severe β‐TM and healthy control children. The functional connectivity found from CPM, indicating the cognitive function, is largely overlapped with the impaired functional connectivity obtained from NBS, implying that the impaired FCs actually impaired the cognitive ability.

Techniques such as the transverse relaxation rate (Cheung et al., 2011), susceptibility‐weighted imaging (SWI) (Qiu, Chan, Chan, Ha, and Khong, 2010), and quantitative susceptibility mapping (QSM) (Qiu et al., 2014) in brain magnetic resonance imaging (MRI) can be used for in vivo and quantitative assessments of cerebral iron levels. Previous studies using these methods have reported iron overload and deposition in the motor, temporal, and subcortical gray matter areas (Manara et al., 2019; Metafratzi et al., 2001; Schweitzer et al., 2015), such as the cerebellum. These findings are consistent with the impaired hub in functional connectivity identified in our study, further supporting the hypothesis of iron overload in these areas. Future research could include more direct investigations of iron content overload using modalities like QSM and SWI to substantiate these findings.

### 4.4 Functional Connectivity Mediates Hematological Abnormalities and Cognitive Decline

This study uses structural equation modeling (SEM) to demonstrate the causal effects of hematological factors on cognitive impairments in β‐TM patients (Liu et al., 2024). Specifically, brain connectivity identified by CMP during the scan mediated the relationship between hematological metric scores (e.g., hemoglobin levels and red blood cell distribution width) and WIS scores (Figure 5). This may suggest that hematological abnormalities in β‐TM patients negatively impact cognitive function through alterations in the brain's functional architecture. However, SEM did not find significant direct evidence of a relationship between hematological metric scores and WIS scores. The underlying pathophysiology through which these hematological pathologies impair cognitive abilities likely involves both external and internal factors, including excessive brain iron accumulation from chronic blood transfusions, ineffective erythropoiesis, and increased intestinal iron absorption, all potentially leading to cognitive impairments.

### 4.5 Data‐driven Approach may Find Novel and Robust Neuromarkers

The data‐driven approach based on Network‐Based Statistics (NBS) and Connectome‐Based Predictive Modeling (CPM) employed in this study identified novel connections used for group comparison and predicting cognitive test outcomes. Previous hypothesis‐driven approaches (Garcia‐Garcia et al., 2018; Qi et al., 2012) have restricted the selection of regions of interest to brain areas known to be associated with specific cognitive tasks. In contrast, data‐driven feature selection methods (Liu, Amey, Backer, Simon, and Forbes, 2022; Rosenberg et al., 2016; Zalesky et al., 2010), like the one adopted in this study, do not have such limitations. Our approach revealed that the neural mechanisms underlying cognitive task processing in β‐TM children may be affected by brain regions that are not ordinarily considered well‐known regions for cognitively intensive tasks, which might be focused on frontal and parietal cortices of the brain. This finding suggests that specific, yet novel, brain connections may be impaired in the cognitive tasks of interest for patients with β‐TM.

Group‐wise analysis also reveals that the functional connectivity for cognitive tasks using CPM only predicts the WIS scores for β‐TM children but not healthy children (Figure 4). This may suggest that the found network particularly exerts an influence on cognitive ability for β‐TM patients. Additionally, another important question raised is whether altered functional connectivity in β‐TM might not only signal impairment but also reflect potential compensatory or protective mechanisms that buffer against further cognitive decline. While our current dataset does not allow us to disentangle resilience from vulnerability, this is a highly relevant direction for future research. In particular, longitudinal designs could determine whether children with preserved or enhanced connectivity in certain networks—such as frontoparietal or cerebellar circuits—exhibit slower rates of decline or better maintenance of specific cognitive abilities. Similarly, multimodal approaches that combine fMRI with behavioral and metabolic assessments may reveal whether certain network configurations mitigate the detrimental impact of hematological abnormalities on cognition. Identifying such protective neural signatures would have significant translational implications, as they could inform targeted interventions aimed at strengthening or preserving network integrity in β‐TM children at risk for cognitive impairment.

### 4.6 Limitations and Future Directions

This study has several limitations. First, the cross‐sectional design of our study allows us to identify associations between brain connectivity and cognition, but it does not permit causal inferences. Future longitudinal studies are needed to track these changes over time. Second, while we identified functional connectivity detrimental to cognitive performance, the underlying mechanisms remain unclear, particularly how iron accumulation affects functional connectivity. Future studies should consider in vivo and quantitative assessments of cerebral iron levels using techniques like transverse relaxation rate, susceptibility‐weighted imaging (SWI), and quantitative susceptibility mapping (QSM) in brain MRI. Third, due to the scarcity of β‐TM data, the findings in this study lack external validation. Additionally, our failure to collect blood samples from the control group limits our ability to compare hematological metrics between β‐TM patients and healthy individuals. This oversight hinders our analysis of how differences in blood composition, such as iron levels and red blood cell counts, might distinguish β‐TM patients from controls. The absence of hematological metrics from control subjects restricts our understanding of the overall influence of these blood characteristics on cognitive functions. Our data‐driven method also has limitations. It is challenging to clarify the role of identified functional networks or brain regions when their original functions are not associated with the target cognitive function. Furthermore, our use of a total cognitive score provides a global measure of impairment, but future studies employing methods like Canonical Correlation Analysis (CCA) could offer more fine‐grained insights by linking specific brain networks to distinct cognitive subdomains. Additionally, the absence of formal power analysis represents a limitation of this study. The relatively small cohort may compromise statistical power. The modest sample size may also inflate some reported effect sizes, particularly given the high‐dimensional feature space, leading to a higher false positive rate for some results (Button et al., 2013). Future studies should aim to replicate these findings with larger sample sizes.

## Author Contributions

**S. X**. performed the research, analysed the data and wrote the paper. **Y. Z**. analysed the data, and wrote the paper; **Y. L**. prepared the software and analysed the data; **X. L**. critically reviewed the manuscript; **S. L**. collected the data; **X. W**. critically reviewed the manuscript; **J. W**. critically reviewed the manuscript; **M. L**. designed the research study, supervised the study and critically reviewed the manuscript; **H. Z**. designed the research study, supervised the study, critically reviewed the manuscript and provided the funding.

## Conflicts of Interest

The authors declare no conflicts of interest.

## Data Availability

Our native MRI images and code will be made available on request from any qualified investigator who provides a practicable proposal, or for the purpose of replicating procedures and results presented in the current study.
